# How algorithmic popularity bias hinders or promotes quality

**DOI:** 10.1038/s41598-018-34203-2

**Published:** 2018-10-29

**Authors:** Giovanni Luca Ciampaglia, Azadeh Nematzadeh, Filippo Menczer, Alessandro Flammini

**Affiliations:** 1 Indiana University Network Science Institute, Bloomington, Indiana USA; 2 School of Informatics, Computing, and Engineering, Indiana University, Bloomington, Indiana USA

## Abstract

Algorithms that favor popular items are used to help us select among many choices, from top-ranked search engine results to highly-cited scientific papers. The goal of these algorithms is to identify high-quality items such as reliable news, credible information sources, and important discoveries–in short, high-quality content should rank at the top. Prior work has shown that choosing what is popular may amplify random fluctuations and lead to sub-optimal rankings. Nonetheless, it is often assumed that recommending what is popular will help high-quality content “bubble up” in practice. Here we identify the conditions in which popularity may be a viable proxy for quality content by studying a simple model of a cultural market endowed with an intrinsic notion of quality. A parameter representing the cognitive cost of exploration controls the trade-off between quality and popularity. Below and above a critical exploration cost, popularity bias is more likely to hinder quality. But we find a narrow intermediate regime of user attention where an optimal balance exists: choosing what is popular can help promote high-quality items to the top. These findings clarify the effects of algorithmic popularity bias on quality outcomes, and may inform the design of more principled mechanisms for techno-social cultural markets.

## Introduction

Cultural markets, such as social media, the entertainment industry, and the world of fashion are known for their continuous rate of innovation and inherent unpredictability. Success of individual actors (e.g., artists) or products (e.g., songs, movies, memes) is in fact hard to predict in these systems^1–3^, mainly due to the presence of strong social reinforcement, information cascades, and the fact that quality is ultimately predicated on intangible or highly subjective notions, such a beauty, novelty, or virality.

In the absence of objective and readily measurable notions of quality, easily accessible metrics of success – such as the number of downloads of a song, or the number of social media followers of an individual – are often taken as input for future recommendations to potential consumers. Popularity and engagement metrics are intuitive and scalable proxies for quality in predictive analytics algorithms. As a result, we are exposed daily to content that is biased to some degree toward popularity, from bestseller lists to search engine results and from trending videos to engaging social media posts^4^.

The usefulness of such rankings is predicated on the *wisdom of the crowd*^5^: high-quality choices will gain early popularity, and in turn become more likely to be selected because they are more visible. Furthermore, knowledge of what is popular can be construed as a form of social influence; an individual’s behavior may be guided by choices of peers or neighbors^6–12^. These mechanisms imply that, in a system where users have access to popularity or engagement cues (such as ratings, number of views, likes, and so on), high-quality content will “bubble up” and allow for a more cost-efficient exploration of the space of choices. This is such a widely shared expectation that it has become routine for social media and e-commerce platforms to highlight popular and trending items. This is also reminiscent of herd behavior in financial markets, where the actions of previous investors carry information, and thus it is rational to adopt them^13^.

Popularity based metrics, however, can bias future success in ways that do not reflect, or worse, that hinder quality. This can happen in different ways. First, lack of independence and social influence among members of the crowd – as that implicitly induced by the availability of rankings – severely undermines the reliability of the popularity signals^10^. Second, engagement and popularity metrics are subject to manipulation, for example by fake reviews, social bots, and astroturf^14,15^.

Popularity bias can have more subtle effects. In search engines, the use of popularity in ranking algorithms was alleged to impede novel content from rising to the top, although such an entrenchment effect was shown to be mitigated by diverse user queries^16^. In social media, some memes inevitably achieve viral popularity in the presence of competition among networked agents with limited attention, irrespective of quality^2^, and the popularity of memes follows a power-law distribution with very heavy tails^17^. Mechanisms such as unfriending and triadic closure facilitate the formation of homogeneous “echo chambers”^18^ or “filter bubbles”^19^ that may further distort engagement metrics due to selective exposure.

Even in the absence of engineered manipulation or social distortion, quality is not necessarily correlated with popularity. Consumers face a trade-off between performing cognitively expensive but accurate assessments based on quality and cognitively cheaper but less accurate choices based on popularity. Adler has shown that the cost of learning about quality will lead to “stars” with disproportionate popularity irrespective of differences in quality^20^. Such trade-offs are common in social learning environments^21^. Salganik *et al*. created a music-sharing platform to determine under which conditions one can predict popular musical tracks^12^. The experiments showed that in the absence of popularity cues, a reliable proxy for quality could be determined by aggregate consumption patterns. However, popularity bias – for example when users were given cues about previous downloads of each track – prevented the quality ranking from being recovered. By influencing choices, popularity bias can reinforce initial fluctuations and crystallize a ranking that is not necessarily related to the inherent quality of the choices^22^. This can happen even in the absence of explicit social signals, if the observed ranking is biased by popularity^23^. Similar results have been found in other studies^8,24–26^ and have spurred a renewed interesting in the topic of predictability in cultural markets. Idealized multinomial logit models have been used to understand the behavior of social influence. Van Hentenryck *et al*.^27^ studied a model of trial-offer markets to analyze the effect of social influence on market predictability. In this model, users chose from a list of items ranked by quality rather than popularity; this modification makes the market predictable and aligns popularity and quality. Empirical tests of specific presentation policies combining quality and popularity do suggest that uncertainty can be reduced in this way^28,29^. Finally, Becker *et al*.^30^ addressed the question of which network structure is most conducive to the wisdom of the crowd when people are influenced by others.

Another line of research that bears some connection to the present work is that of network growth models. Bianconi and Barabási^31^ incorporated a notion of fitness in the preferential attachment model^32^. This approach provides one way to combine choices based on popularity and quality, but not a way to explore the effect of different mixtures between the two ingredients.

The conditions in which popularity bias promotes or hinders quality content have not been systematically explored. Here we do so by studying an idealized cultural market model in which agents select among competing items, each with a given quality value. A parameter regulates the degree to which items are selected on the basis of their popularity rather than quality. We find that this popularity bias yields a rich behavior when combined with the cognitive cost of exploring less popular items. As we shall see, popularity bias tends to hinder quality in general; but for a critical exploration cost, some popularity bias results in maximal average quality.

## Results

Our model considers a fixed number *N* of items. These represent transmissible units of information, sometimes referred to as *memes*^33^, such as music tracks, videos, books, fashion products, or links to news articles. Items are selected sequentially at discrete times. Each item *i* has an *intrinsic quality* value *q*_*i*_ drawn uniformly at random from [0, 1]. Quality is operationally defined as the probability that an item is selected by a user when not exposed to the popularity of the item. The *popularity* of item *i* at time *t*, *p*_*i*_(*t*), is simply the number of times *i* has been selected until *t*. At the beginning each item is equally popular: *p*_*i*_(0) = 1, *i* = 1 … *N*.

At each time step, with probability *β*, an item is selected based on its popularity. All items are first ranked by their popularity, and then an item is drawn with probability proportional to its rank raised to some power:

1 $${P}_{i}^{pop}\,(t)=\frac{{r}_{i}{(t)}^{-\alpha }}{\sum _{j=1}^{N}\,{r}_{j}^{-\alpha }(t)}$$

where the rank *r*_*i*_(*t*) is the number of items that, at time *t*, have been selected at least as many times as *i*. The exponent *α* regulates the decay of selection probability for lower-ranked items. This schema is inspired by the *ranking model*, which allows for the emergence of scale-free popularity distributions with arbitrary power-law exponents^34^; it is consistent with empirical data about how people click search engine results^16^ and scroll through social media feeds^35^. This model could accurately capture aggregate behavior even if individuals followed different selection schemes^36^.

Alternatively, with probability 1 − *β*, an item is drawn with probability proportional to its quality:

2 $${P}_{i}^{qual}=\frac{{q}_{i}}{\sum _{j=1}^{N}\,{q}_{j}}.$$

After an item *i* has been selected at time *t*, we update its popularity (*p*_*i*_(*t* + 1) = *p*_*i*_(*t*) + 1) and the ranking. Two items have the same rank *r* if they have been selected the same number of times. If *n* item are all at the same rank *r*, then the next rank is *r* + *n*.

The model has two parameters: *β* regulates the importance of popularity over quality and thus represents the *popularity bias* of the algorithm. When *β* = 0, choices are entirely driven by quality (no popularity bias). When *β* = 1, only popularity choices are allowed, yielding a type of Polya urn model^37^. The parameter *α* can be thought of as an *exploration cost*. A large *α* implies that users are likely to consider only one or a few most popular items, whereas a small *α* allows users to explore less popular choices. In the limit *α* → 0, the selection no longer depends on popularity, yielding the uniform probability across the discrete set of *N* items. Another way to think about the parameter *α* is as inversely related to *attention:* low *α* means that users have sufficient attention to consider all items (high attention), while high *α* means that users have attention for only a limited number of items (low attention).

At equilibrium, after a large number of selection steps *T*, we characterize two properties of the distribution of popularity ${\{{p}_{i}\}}_{i=1}^{N}$ with respect to the intrinsic quality distribution ${\{{q}_{i}\}}_{i=1}^{N}$. For brevity, we pose *p*_*i*_ = *p*_*i*_(*T*) here. The first quantity we measure is the *average quality* $\bar{q}={\sum }_{i\mathrm{=1}}^{N}\,{p}_{i}{q}_{i}/{\sum }_{i\mathrm{=1}}^{N}\,{p}_{i}$ and the second property *τ* is the *faithfulness* of the algorithm, i.e., the degree to which quality is ref lected in popularity. We quantify faithfulness using Kendall’s rank correlation between popularity and quality^38^. The question we ask is whether it is possible to leverage some popularity bias to obtain a higher average quality, even at the cost of decreasing the algorithm’s faithfulness.

We can derive the values of both properties in the extreme cases of popularity bias. When *β* = 0, selections are made exclusively on the basis of quality and therefore one expects *p*_*i*_ → *q*_*i*_ as *T* → ∞. The rankings by quality and popularity are therefore perfectly aligned, and *τ* = 1. In the limit of large *N* we can make a continuous approximation $\bar{q}\to {\int }_{0}^{1}\,{q}^{2}dq/{\int }_{0}^{1}\,q\,dq=2/3$. When *β* = 1, quality never enters the picture and any permutation of the items is an equally likely popularity ranking, which translates into *τ* = 0. Also *p*_*i*_ → 1/*N* and in the continuous approximation $\bar{q}\to {\int }_{0}^{1}\,q\,dq=1/2$.

What happens for intermediate values of popularity bias is harder to predict due to the role played by ranking, especially for high values of *β* where initial fluctuations can be strongly enhanced by the popularity-based ranking algorithm. Let us nevertheless try to derive $\bar{q}$ at equilibrium by discretizing *q* ≈ *k*/*N* for *k* = 1 … *N*:

3 $$\bar{q}\approx \frac{\sum _{k=1}^{N}\,\frac{k}{N}[\mathrm{(1}-\beta ){P}_{k}^{qual}+\beta {P}_{k}^{pop}]}{\sum _{k=1}^{N}\,\mathrm{(1}-\beta ){P}_{k}^{qual}+\beta {P}_{k}^{pop}}.$$

Using Eq. 2 we can write the first probability term as

4 $${P}_{k}^{qual}\approx \frac{k/N}{\sum _{j=1}^{N}\,j/N}=\frac{2k}{N(N+\mathrm{1)}}.$$

For the second term of the probability, we assume that the rank will converge to a value that depends on *β*. In the extreme *β* = 0, the rank of the item with quality *k*/*N* should be narrowly peaked around *r*_*k*_ = *N* − *k* + 1, yielding the top rank when *k* = *N* (maximum quality) and the bottom rank when *k* = 1 (lowest quality). In the extreme *β* = 1, quality plays no role, and therefore all ranks are equally probable, no matter the value of the item quality. For intermediate *β* we interpolate between these extremes, assuming that *r*_*k*_ is uniformly distributed between two limits ${r}_{k}^{min}=1+(N-k\mathrm{)(1}-{\beta }^{\gamma })$ and ${r}_{k}^{max}=N-(k-\mathrm{1)(1}-{\beta }^{\gamma })$. The heuristic parameter *γ* captures how effectively the perfect information produced (for ranking) by quality-based choices is preserved as *β* increases. From these assumptions and Eq. 1 we obtain

5 $${P}_{k}^{pop}\approx \frac{1}{{r}_{k}^{max}-{r}_{k}^{min}}\,\sum _{{r}_{k}={r}_{k}^{min}}^{{r}_{k}^{max}}\,\frac{{r}_{k}^{-\alpha }}{\sum _{j=1}^{N}\,{j}^{-\alpha }}.$$

**Figure 1:**
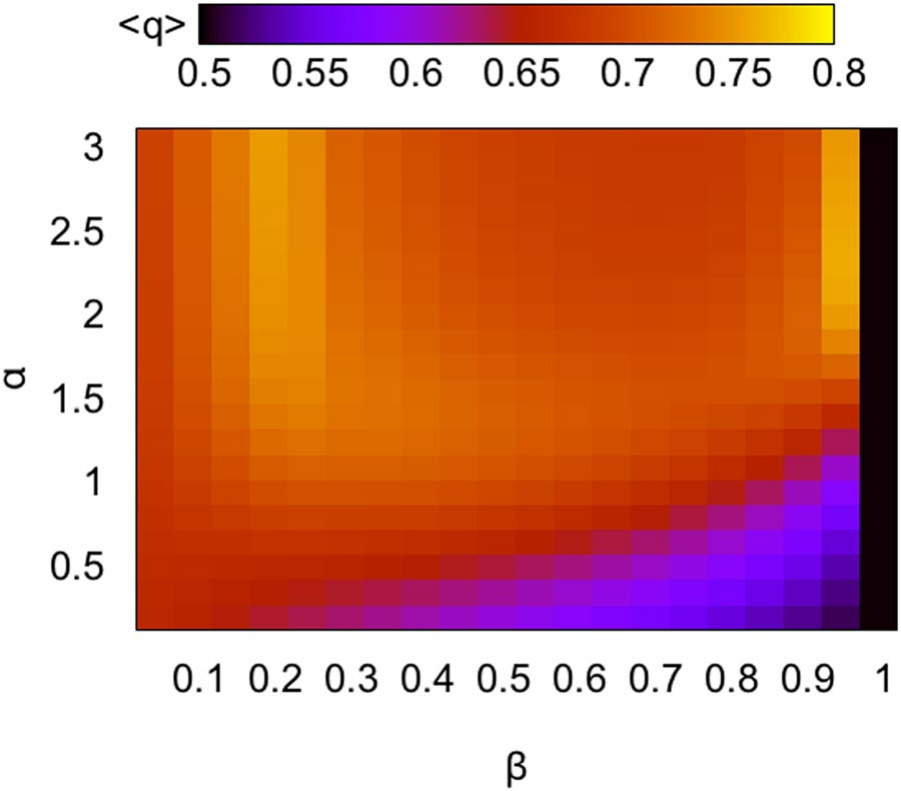
Predicted average quality at equilibrium. Heatmap of average quality $\bar{q}$ as a function of *α* and *β*, based on numerical solution of equilibrium condition derived in the text.

We plugged the approximations from Eqs 4 and 5 into Eq. 3 and solved numerically using *γ* = 4 to obtain the equilibrium prediction shown in Fig. 1. The analysis suggests a non-trivial behavior of the system, with a maximum in average quality $\bar{q}$ for intermediate values of *β* when *α* is not too small and another maximum for high *β* and *α*.

Given the strong assumptions in our derivation and the consequent uncertainty of the solution, let us turn to simulations for a more reliable analysis of the model’s behavior. We vary *β* systematically in [0, 1] and consider different values of *α* between 0 and 3. We simulate 100 realizations for each parameter configuration. In each realization we perform *T* = 10^8^ selections among *N* = 1000 items using Eqs 1 and 2 and store the final popularity values.

**Figure 2:**
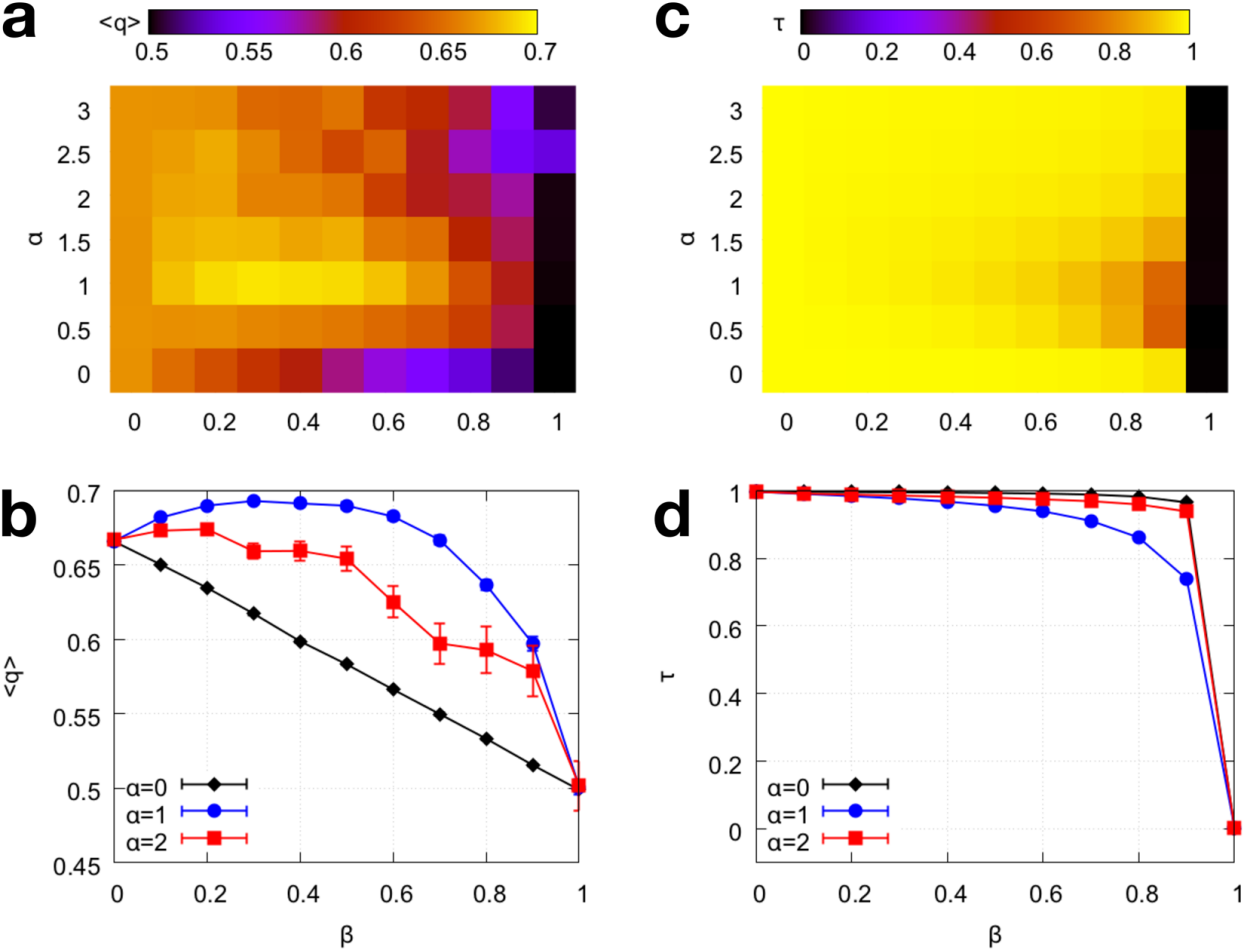
Effects of popularity bias on average quality and faithfulness from model simulations. (**a**) Heatmap of average quality $\bar{q}$ as a function of *α* and *β*, showing that popularity bias can either hinder or promote average quality depending on exploration cost. (**b**) The location of the maximum $\bar{q}$ as a function of *β* depends on *α*, here shown for *α* = 0, 1, 2. When *α* = 1 an intermediate amount of popularity bias is optimal. (**c**) Faithfulness *τ* of the algorithm as a function of *α* and *β*. (**d**) *τ* as a function of *β* for the same three values of *α*. Standard errors are shown in panels (**b**,**d**) and are smaller than the markers if not visible.

The dependence of the average quality $\bar{q}$ on the popularity bias *β* and exploration cost *α* is shown in Fig. 2(a,b). We observe that the predictions for *β* = 0 ($\bar{q}$ = 2/3) and *β* = 1 ($\bar{q}$ = 0.5) were correct. For intermediate popularity bias, the derived solution predicted that the optimal amount of popularity bias depends non-trivially on the exploration cost; however the simulation results provide a more accurate picture. When attention is abundant and the exploration cost is small (*α* < 1), popularity bias only hinders quality; the best average quality is obtained for *β* = 0. In the opposite extreme of very limited attention, when popularity-based choices are strongly focused on the top-ranked items (*α* = 3), the trend is more noisy but popularity bias tends to hinders quality; the optimal value of $\bar{q}$ is again attained for *β* = 0. The most interesting behavior is observed at a critical regime of attention, when the exploration cost is around *α* = 1. Here, the optimal value of $\bar{q}$ is attained for an intermediate value of popularity bias $\hat{\beta }={\rm{\arg }}\,{{\rm{\max }}}_{\beta }\,\bar{q}(\beta )\approx 0.3$. When *β* is smaller, the system may not be taking advantage of quality signals crowdsourced from other users. When *β* is higher, the system may be amplifying random initial fluctuations in popularity.

In Fig. 2(c,d) we show the behavior of faithfulness *τ* as a function of *α* and *β*. We observe that *τ* = 1 for *β* = 0, as predicted. Popularity bias always hinders the algorithm’s faithfulness, however the effect is small as long as *β* is not too large. This suggests that in the regime where popularity bias improves quality on average, there is a small price to be paid in terms of over-represented low-quality items and under-represented higher-quality items. In general, the algorithm can retain faithfulness in the presence of moderate popularity bias. Near *α* = 1 we observe a degradation in faithfulness as *β* grows larger, as low-quality items are wrongly picked up and become popular. This explains the degradation in average quality. But in general, *τ* remains relatively high over a wide range of popularity bias values. We observe a sharp transition to the predicted value *τ* = 0 at *β* = 1, when quality no longer plays a role and popularity merely amplifies random fluctuations.

**Figure 3:**
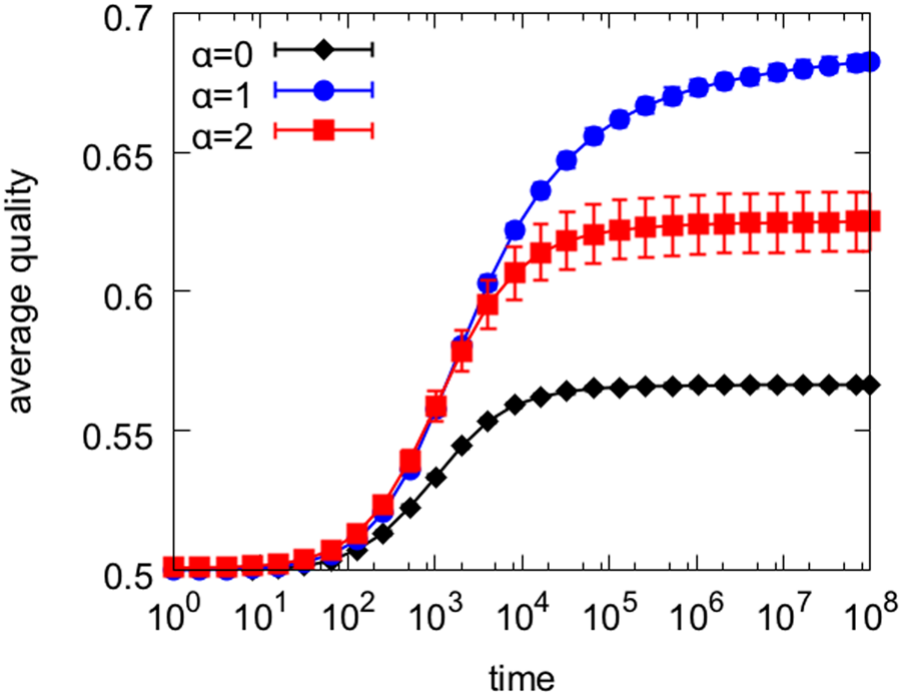
Temporal evolution of average quality. Average quality $\bar{q}$ is traced over time for *β* = 0.6 and different values of exploration cost. Error bars represent standard errors across runs. Compared to the optimal case *α* = 1, with more exploration (*α* = 0) the popularity bias just adds noise, and with less exploration (*α* = 2) it causes the system to converge prematurely to sub-optimal quality.

For a given value of *β*, if *α* is low, the popularity bias hinders quality because it fails to enhance the signal provided by the quality-based choices, which are supported by exploration. To understand why quality is also hindered by the popularity bias when *α* takes higher values, consider the evolution of the average quality in simulations of the model for different values of *α*, shown in Fig. 3. By focusing only on the top ranked items (*α* = 2), the system converges prematurely to a sub-optimal ranking, producing lower quality on average. In other words, with insufficient exploration the popularity bias risks enhancing initial noise rather than the quality-based signal. With more exploration (*α* = 1), $\bar{q}$ continues to grow. The premature convergence to sub-optimal ranking caused by excessive popularity bias is also reflected in the increased variance of the average quality across runs of the model (larger error bars). This is consistent with the increase in variance of outcomes observed in other studies^12,22^.

## Discussion

Cultural markets like social media and the music and fashion industry account for multi-billion dollar businesses with worldwide social and economic impact^1^. Success in these markets may strongly depend on structural or subjective features, like competition for limited attention^2,35^. The inherent quality of cultural products is often difficult to establish, therefore relying on measurable quantitative features like the popularity of an item is hugely advantageous in terms of cognitive processing and scalability.

Yet, previous literature has shown that recommending already popular choices can be detrimental to the predictability and overall quality of a cultural market^12^. This left open the question of whether there exist situations in which a bit of popularity bias can help high-quality items bubble up in a cultural market.

In this paper we answered this question using an extremely simplified abstraction of cultural market, in which items are endowed with inherent quality. Our results show that optimizing the average quality of consumed items requires a careful tuning of quality- and popularity-based choices that depends on the focus on the most popular items. Popularity bias hinders average quality when users are capable of exploring many items, as well as when they only consider very few top items due to scarce attention. Critically, we found an intermediate regime of mild exploration cost in which some popularity bias is good, but too much is bad.

The model could be extended in many directions, for example assuming a population of networked agents with heterogeneous parameters. However, our approach leads to very general findings about the effects of popularity bias. While we confirmed that such a bias can distort assessments of quality, the scenario emerging from our analysis is richer than suggested by prior literature. First, it is possible to maintain a good correspondence between popularity and quality rankings of consumed items even when our reliance on popularity for our choices is relatively high. Second, one can leverage the wisdom of the crowd in the presence of limited attention, or let users make their own decisions when they are able to explore many items.

From a normative perspective, our results provide a recipe for improving the quality of content in techno-social cultural markets driven by engagement metrics, such as social media platforms. It is possible in these systems to estimate the exponent *α* empirically, by measuring the probability that a user engages with an item as a function of the item’s position in the feed. Given a statistical characterization (e.g., average or distribution) of the exploration cost, the bias *β* of the ranking algorithm could be tuned to maximize expected average quality.

These findings are important because in our information-flooded world we increasingly rely on algorithms to help us make consumption choices. Platforms such as search engines, shopping sites, and mobile news feeds save us time but also bias our choices. Their algorithms are affected by and in turn affect the popularity of products and information, and ultimately drive what we collectively consume in ways that we do not entirely comprehend. It has been argued, for example, that the engagement bias of social media ranking algorithms is partly responsible for the spread of low-quality content over high-quality material^39^. Evaluating such a claim is challenging, but the present results may lead to a better understanding of algorithmic bias.
